# Cushing syndrome of different etiologies - cardiometabolic complications, venous thromboembolic events and mortality: data from ERCUSYN Krakow database

**DOI:** 10.3389/fendo.2026.1785054

**Published:** 2026-02-25

**Authors:** Mari Minasyan, Aleksandra Gamrat-Żmuda, Agata Bryk-Wiązania, Wiktoria Suchy, Anna Bogusławska, Ewelina Rzepka, Beata Piwońska-Solska, Katarzyna Majka, Alicja Hubalewska-Dydejczyk, Elena Valassi, Aleksandra Gilis-Januszewska

**Affiliations:** 1 Chair and Department of Endocrinology, Jagiellonian University Medical College, Kraków, Poland; 2 Students’ Scientific Circle of the Department of Endocrinology, Jagiellonian University Medical College, Kraków, Poland; 3 Endocrinology Department, Hospital Germans Trias i Pujol, Badalona, Barcelona, Spain; 4 Universitat Internacional de Catalunya (UIC), Barcelona, Spain

**Keywords:** Cushing, ERCUSYN, hypercortisolemia, pituitary, ectopic, adrenal, venous thromboembolic events

## Abstract

**Introduction:**

Cushing syndrome (CS) as a state of prolonged cortisol excess is associated with multiple complications that contribute to increased mortality in affected patients.

**Materials and methodology:**

This retrospective study presents data on etiology, demographic features, baseline cardiometabolic comorbidities, venous thromboembolic events and mortality of 214 consecutive CS patients from a single tertiary endocrinology center in Poland, a part of the European Register on Cushing’s Syndrome (ERCUSYN). The group was predominated by pituitary CS (53%, PIT-CS), followed by adrenal CS (25%, ADR-CS) and ectopic CS (22%, ECT-CS). Statistica 13.0 was used to perform data analysis. Statistical significance was settled for a p-value ≤0.05.

**Results:**

The PIT-CS group was significantly younger than others. The PIT-CS and ADR-CS groups were predominated by women, contrary to the ECT-CS group, predominated by men. At the baseline, respectively 80%, 78%, and 66% of patients presented hypertension, dyslipidemia, and glucose metabolism impairments. Ischemic heart disease and heart failure were significantly more prevalent among ECT-CS. Venous thromboembolic events were present among 6% of patients. Overall mortality rate was 18%, and was higher in males than females (30% vs 15%; p<0.05), and was the highest in ECT-CS group (62%). The most common cause of death was tumor progression (55%) and infectious disease (26%).

**Conclusions:**

CS patients from our study presented a high number of comorbidities and high mortality rate. Some of the results were convergent with reports of the entire ERCUSYN database and other studies, while other results differed from the data reported in the literature.

## 1 Introduction

Cushing syndrome (CS) is a state of prolonged cortisol excess, mostly due to exogenous steroids use. Endogenous CS occurs with the frequency of 2–8 people per million annually (1, 2). It is predominated by ACTH-dependent CS, mainly originating in pituitary corticotroph adenoma (Cushing Disease), and less frequently caused by ectopic adrenocorticotropic (ACTH) or corticotropin releasing hormone (CRH) production (2–4). Autonomous cortisol production by adrenal tumors or adrenal hyperplasia represents another cause of endogenous CS (2–4). CS is associated with cardiovascular, thrombotic, metabolic, infectious, musculoskeletal and psychiatric complications, all of which increase mortality rate and impair quality of life among these patients (1, 5–7). The clinical picture varies individually, which may probably be associated with glucocorticoid receptor polymorphism and its different sensitivity for cortisol stimulation (8). Early CS recognition and proper identification of CS etiology is crucial for preventing symptoms and progression of complications. Some CS features may overlap with features existing in non-CS patients who have metabolic and cardiovascular comorbidities (1, 9, 10). Therefore, a delay of diagnosis can be considerably long, which leads to a higher rate of CS complications, poor quality of life and decreased life expectancy. Another relevant issue regards the fact that some of the comorbidities and complications of CS may persist even after successful CS treatment (11–15). Thus, CS patients require interdisciplinary and long-term health care, even after successful hypercortisolemia treatment.

The European Registry on Cushing’s syndrome (ERCUSYN) is a web-based, multicenter, observational study, which collects data on the symptomatology, diagnosis, epidemiology, comorbidities and management in patients with CS (3, 16–25). The last report on clinical and etiological presentation of patients from the ERCUSYN database, is the report from 2022, which involved 1564 patients (ERCUSYN 2022) (3).

The present study analyzes CS patients from Krakow ERCUSYN database.

### 1.1 Aim

The objective of this study was to characterize baseline cardiometabolic comorbidities, venous thromboembolic events and mortality among CS patients of different etiologies in a single tertiary endocrinology center, a part of ERCUSYN.

## 2 Materials and methods

The current study is a retrospective analysis of baseline data of the consecutive patients from the ERCUSYN Krakow database, who were diagnosed between January 1997 and June 2025. Data extraction from the registry was performed in September 2025.

Patients were classified into the following three major groups depending on the diagnosis: pituitary-dependent CS (PIT-CS), adrenal-dependent CS (ADR-CS), and CS from ectopic source (ECT-CS). Etiological classification was based on histopathological examination results. If such documentation was unavailable, hypercortisolemia resolution after surgical treatment confirmed the origin of the CS. In patients who were not operated on, etiological classification was found in imaging and biochemical test results. Diagnostic interpretations of examinations that led to the diagnosis of Cushing syndrome were based on international guidelines (1). Patients’ clinical and demographic features, radiological imaging findings, venous thromboembolic events (VTE), cardiovascular, and metabolic comorbidities, were assessed at the time of CS diagnosis. Additionally, we followed the patients for occurrence and cause of death.

Obesity was diagnosed for body mass index (BMI) >/=30kg/m2, overweight for BMI 25-29.9kg/m2, underweight for BMI <18.5 kg/m2. Hypertension was defined as systolic blood pressure ≥140 and/or diastolic BP ≥ 90 mmHg and/or treatment with antihypertensive agents. Hypertension exacerbation was defined as worsening of blood pressure control requiring intensification of antihypertensive therapy. Diabetes mellitus (DM) was defined as fasting blood glucose (FBG) >/=126 mg/dL, 2-hour plasma glucose >/= 200 mg/dL during a 75g oral glucose tolerance test, glycated hemoglobin (HBA1C) >/= 6.5%, or random blood glucose >/= 200 mg/dL in the presence of classic symptoms of hyperglycemia. Prediabetes included impaired fasting glucose (IFG), defined as FBG 100–125 mg/dL, and impaired glucose tolerance (IGT), defined as 2-hour plasma glucose 140–199 mg/dL during a 75g oral glucose tolerance test. DM or prediabetes deterioration was defined as worsening of glycemic control requiring intensification of treatment.

Statistica 13.0 was used to perform data analysis. The data wasn’t normally distributed. Data was presented as a median with interquartile range. Comparisons between CS etiologic groups were conducted by the Kruskal-Wallis test for numerical variables, and the chi squared test for categorical variables. The Kaplan-Meier curve was provided to compare the rate of survival in different etiological groups. A Bonferroni correction was used in all of the comparisons. Statistical significance was settled for a p-value ≤0.05. For each statistical comparison, we considered only patients with available specific pieces of information needed for the analyzes (e.g. information regarding the presence/absence of diabetes, information regarding the presence/absence of hypertension, information regarding the result of pituitary imaging). In case there was not such information available in the patient’s medical documentation, the individual was not included in the analysis. The information regarding the number of patients included in the analyzes was given in the particular tables.

The ERCUSYN Krakow study was approved by the local ethics committee.

## 3 Results

### 3.1 CS etiology

At the time of data extraction, ERCUSYN Krakow database included 214 patients (53% PIT-CS, 25% ADR-CS, 22% ECT-CS) (**Table 1**).

**Table 1:** Cushing syndrome (CS) etiology presentation in European Registry on Cushing’s syndrome (ERCUSYN) Krakow database.

Total group, n	PIT-CS, n(%)	ADR-CS, n(%)	ECT-CS, n(%)
214	114 (53)	53 (25)	47 (22)

PIT-CS, pituitary CS; ADR-CS, adrenal CS; ECT-CS, ectopic CS.

#### 3.1.1 PIT-CS

Pituitary magnetic resonance imaging (MRI) results were available for 114 of PIT-CS patients (100%). Pituitary microadenoma was present in 59% (67) of patients and pituitary macroadenoma was present in 32% (37) of patients. In 9% (10) of patients, pituitary lesion was not visualized by MRI. In 26% (30) of patients with PIT-CS (23 patients with microadenoma and 7 patients without visualized pituitary lesion), inferior petrosal sinus sampling (IPSS) has been performed to confirm Cushing disease (CD).

Histopathological results were available in 41 patients (36%) with PIT-CS. The most prevalent finding was densely granulated corticotroph pituitary neuroendocrine tumor (PitNET, 68%), followed by sparsely granulated PitNET (15%) and PitNET with Crooke cells (10%). Among 7% of patients, no pituitary tumor tissue was found despite successful surgery.

#### 3.1.2 ADR-CS

Adrenal lesions in ADR-CS were equally commonly represented by single left side adrenal adenoma present in 19 (36%) patients and bilateral adrenal adenomas present in 19 (36%) patients. Right adrenal lesion was present among 24.5% of patients. The least prevalent was multiple left side adrenal adenomas (3.5%).

The histopathological result was available in 33 patients (62%) with ADR-CS. It was predominated by adrenal cortex adenoma in 17 (52%) patients, followed by adrenal hyperplasia (18%), adrenal oncocytoma (15%), concomitant presence of adenoma and oncocytoma (6%), and adenoma with myelolipomatous transformation (6%). The least prevalent was black adrenal cortex adenoma (3%).

#### 3.1.3 ECT-CS

Histopathological reports were available for 38 ECT-CS patients. ECT-CS was mainly predominated by small cell lung cancer, which was present in 9 patients (20%, SCLC), followed by lung carcinoid in 7 (16%) patients. The least prevalent findings included breast cancer in 1 patient (2%), uterine clear cell cancer in 1 patient (2%), gastric adenocarcinoma in 1 patient (2%), intestinal neuroendocrine tumor (NET) in 1 patient (2%), gastric NET in 1 patient (2%), gastric neuroendocrine cancer in 1 patient (2%), lung adenocarcinoma in 1 patient (2%), maxillary sinus papilloma in 1 patient (2%) and esthesioneuroblastoma in 1 patient (2%). 5 patients (11%) with ECT-CS did not have an identified source of ACTH overproduction.

**Figure 1:**
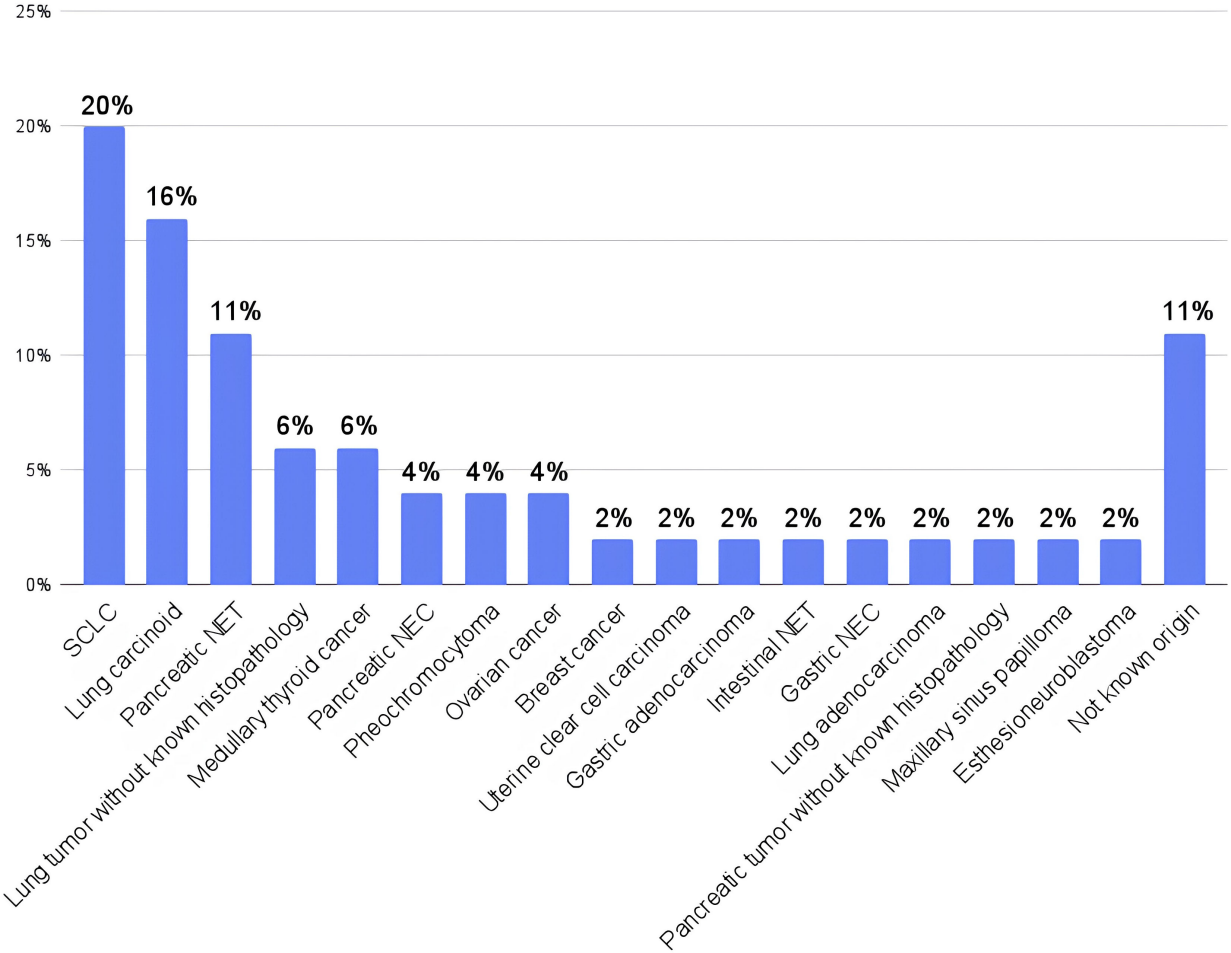
Ectopic Cushing syndrome- histopathology presentation in European Registry on Cushing’s syndrome (ERCUSYN) Krakow database. SCLC, small cell lung cancer; NET, neuroendocrine tumor; NEC, neuroendocrine carcinoma.

The results are presented in **Figure 1**.

### 3.2 General characteristics of the overall population and each etiologic group

The entire CS group was predominated by women (women to men ratio=3:1). The percentage of women in PIT-CS and ADR-CS was higher than in ECT-CS (83%, 87% vs 51%, p<0.001). Women in ECT-CS were significantly older than men (65[62-74] vs 59[44-66] years, p<0.05). No significant differences in BMI were observed between the etiological groups. In total, 27% of patients were actively smoking cigarettes and 14% were ex-smokers. In the overall group, the median delay to diagnosis (the time from the first symptoms to the diagnosis) was 12(5-26) months. This period was shorter in ECT-CS patients as compared with PIT-CS and ADR-CS (2[1-4] vs 16[9-36], 15[7-24] months) patients (p<0.001). The results regarding general characteristics are presented in **Table 2**.

**Table 2:** General characteristics of the overall population and each etiologic group in European Registry on Cushing’s syndrome (ERCUSYN) Krakow database.

Parameters	Total	PIT-CS	ADR-CS	ECT-CS	Statistically significant differences
Age (years)	54(38-65)	46(33-61)	57(42-66)	63(51-70)	***PIT-CS vs ECT-CS ** PIT-CS vs ADR-CS
Age women (years)	54(38-65)	49(36-62)	56(38-66)	65(62-74)	*** ECT-CS vs PIT-CS ** ECT-CS vs ADR-CS
Age men (years)	55(36-64)	36(22-57)	61(54-64)	59(44-66)	* ADR-CS vs PIT-CS * ECT-CS vs PIT-CS
Women	165/214 77%	95/114 83%	46/53 87%	24/47 51%	*** ECT-CS vs PIT-CS *** ECT-CS vs ADR-CS
BMI (kg/m2)	29(25-33) N:189	28(25-33) N:107	30(26-33) N:51	29(24-33) N:31	–
Weight (kg)	77(68-89) N:189	78(68-88) N:107	76(70-87) N:51	75(66-97) N:31	–
Obesity	77/189 40.7%	39/107 36.5%	26/51 51%	12/31 39%	–
Overweight	71/189 37.6%	45/107 42%	17/51 33%	9/31 29%	–
Normal weight	39/189 20.6%	23/107 21.5%	7/51 14%	9/31 29%	–
Underweight	2/189 1.1%	0/107	1/51 2%	1/31 3%	–
Systolic BP (mmHg)	142(130-155) N:183	143(130-154) N:98	140(130-156) N:49	140(122-160) N:36	–
Diastolic BP (mmHg)	87(78-97) N:183	88(79-96) N:98	90(80-98) N:49	80(70-100) N:36	–
Time from first symptoms to the diagnosis (months)	12(5-26) N:183	16(9-36) N:99	15(7-24) N:49	2(1-4) N:35	***ECT CS vs ADR-CS ***ECT-CS vs PIT-CS
Active cigarette smoker	55/202 27%	25/111 23%	20/52 38%	10/39 26%	–
Ex cigarette smoker	29/202 14%	15/111 14%	7/52 13%	7/39 18%	–

PIT-CS, pituitary CS; ADR-CS, adrenal CS; ECT-CS, ectopic CS (*p<0.05, **p<0.01, ***p<0.001).

### 3.3 Metabolic and cardiovascular comorbidities

A total of 66% of patients had glucose tolerance impairment. The prevalence of DM was 54%, IFG 7%, and IGT 6%. In 88% of these patients, impaired glucose tolerance was identified at the time of CS evaluation, or pre-existing disturbances in glucose homeostasis deteriorated.

The ECT-CS group had higher prevalence of DM than other groups (p<0.001).

Overall, 80% of patients had hypertension. Among these patients, 64% had hypertension diagnosed at the time of CS evaluation or experienced deterioration of pre-existing hypertension control.

Ischemic heart disease was present in 25% of CS patients and was more prevalent in ECT-CS than in PIT-CS (32% vs 11%, p<0.01).

Heart failure was present in 19% of CS patients and was more prevalent in ECT-CS than PIT-CS (34% vs 13%, p<0.01).

Stroke was more prevalent in men than women (13% vs 4% p<0.05). The results are presented in **Table 3**.

**Table 3:** Metabolic and cardiovascular comorbidities of the overall population and each etiologic group in European Registry on Cushing’s syndrome (ERCUSYN) Krakow database.

Comorbidities	Total	PIT-CS	ADR-CS	ECT-CS	Statistically significant differences
All patients with glucose tolerance impairment (DM+IFG+IGT)	141/213 66%	72/113 64%	30/53 57%	39/47 83%	**ECT-CS vs ADR-CS *ECT-CS vs PIT-CS
Cases with newly diagnosed or exacerbated impaired glucose tolerance	124/141 88%	65/72 90%	28/30 93%	31/39 79%	–
DM	114/213 54%	53/113 47%	22/53 42%	39/47 83%	***ECT-CS vs ADR-CS ***ECT-CS vs PIT-CS
IFG	14/213 7%	8/113 7%	6/53 11%	0/47	***ECT-CS vs ADR-CS ***ECT-CS vs PIT-CS
IGT	13/213 6%	11/113 10%	2/53 4%	0/47	***ECT-CS vs ADR-CS ***ECT-CS vs PIT-CS
Prediabetes (IFG+IGT)	27/213 13%	19/113 17%	8/53 15%	0	***ECT-CS vs ADR-CS ***ECT-CS vs PIT-CS
Hypertension	169/212 80%	83/112 74%	45/53 85%	41/47 87%	–
Newly diagnosed hypertension/exacerbation among all patients with hypertension	108/169 64%	56/83 67%	30/45 67%	22/41 54%	–
Ischemic heart disease	36/212 25%	12/112 11%	9/53 17%	15/47 32%	**PIT-CS vs ECT-CS
Atrial fibrillation	18/195 9%	7/107 7%	6/52 12%	5/36 14%	–
Dyslipidemia	164/211 78%	86/111 77%	44/53 83%	34/47 72%	–
Stroke	11/191 6%	4/107 4%	4/50 8%	3/34 9%	–
Chronic renal dysfunction	9/197 5%	2/108 2%	4/51 8%	3/38 8%	–
Heart failure	41/212 19%	14/112 13%	11/53 21%	16/47 34%	**PIT-CS vs ECT-CS

PIT-CS, pituitary CS; ADR-CS, adrenal CS; ECT-CS, ectopic CS. DM, diabetes mellitus; IFG, impaired fasting glucose; IGT, impaired glucose tolerance (*p<0.05, **p<0.01, ***p<0.001).

### 3.4 Venous thromboembolic complications

In the overall CS group, the prevalence of VTE complications was 6% (12/201). Seven patients had deep venous thrombosis (DVT), four patients had pulmonary embolism (PE) and one patient presented with both events. Nine patients experienced a VTE event prior to the diagnosis of CS (range, 2–120 months). In three patients, the VTE event led to the diagnosis of CS (i.e. first manifestation of hypercortisolism). The results are presented in **Table 4**.

**Table 4:** Venous thromboembolic events (VTE) in the overall population and each etiologic group in European Registry on Cushing’s syndrome (ERCUSYN) Krakow database.

Type of VTE	Total	PIT-CS	ADR-CS	ECT-CS	Statistically significant differences
Total	12/201 6%	5/112 4%	6/52 12%	1/37 3%	–
DVT	7/12 58.3%	2/5 40%	4/6 67%	1/1 100%	–
PE	4/12 33.3%	2/5 40%	2/6 33%	0 0	–
DVT+PE	1/12 8.3%	1/5 20%	0	0	–
Cases in whom VTE prompted endocrinology referral	3/12 25%	1	1	1	–

PIT-CS, pituitary CS; ADR-CS, adrenal CS; ECT-CS, ectopic CS. DVT denotes deep vein thrombosis; PE denotes pulmonary embolism (*p<0.05, **p<0.01, ***p<0.001).

### 3.5 Mortality

Information on current patients’ status was available for 206 patients. Overall mortality was 18% (38 patients, 26 ECT-CS, 7 PIT-CS, 5 ADR-CS). Overall, men showed higher mortality than women (30% vs 15%; p<0.05).

Mortality was significantly higher in ECT-CS as compared with PIT-CS and ADR-CS (62% vs. 6% and 10%, respectively; p<0.001).

**Figure 2:**
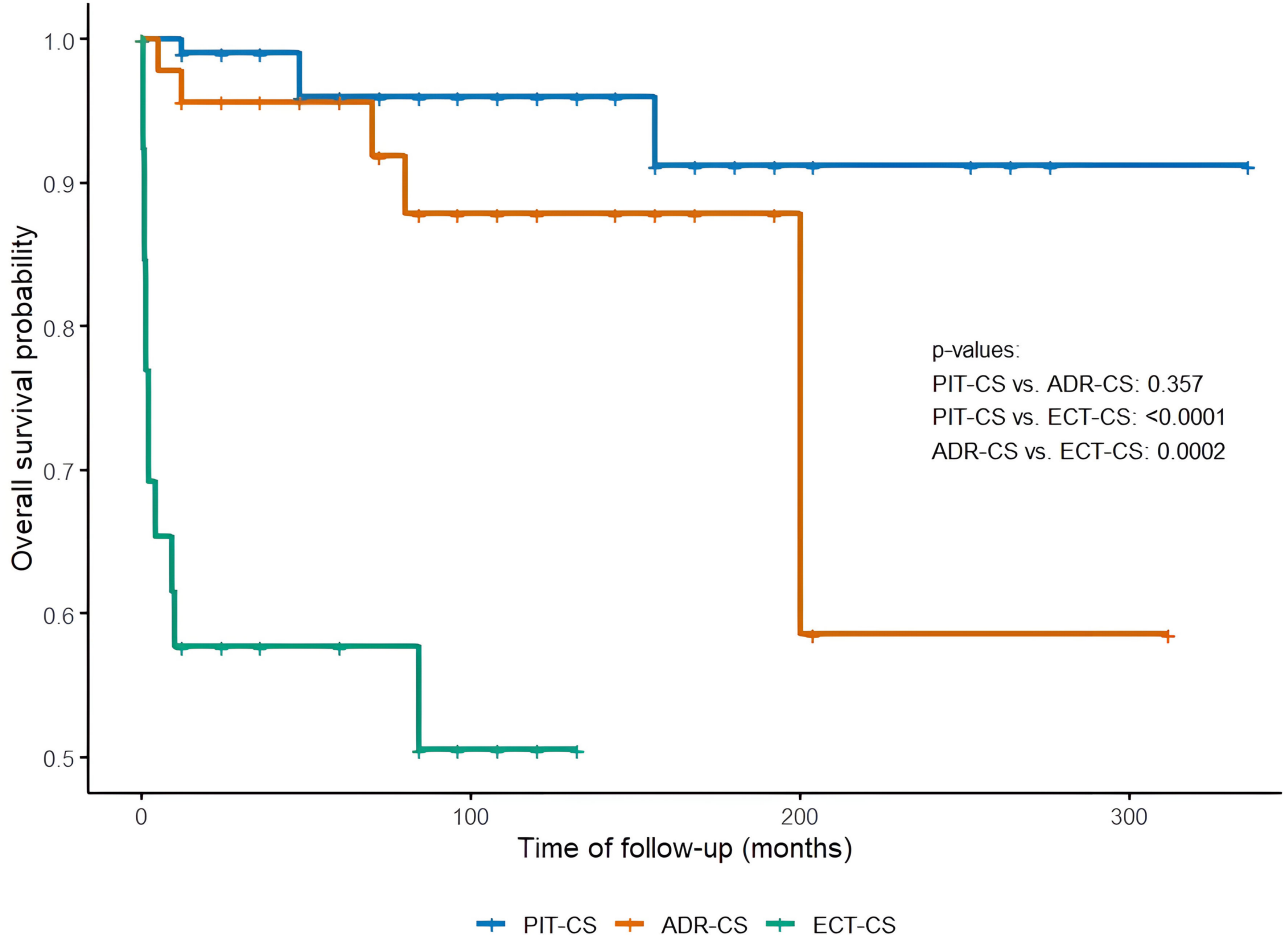
Kaplan-Meier survival curve in each etiologic group in European Registry on Cushing’s syndrome (ERCUSYN) Krakow database. PIT-CS, pituitary CS; ADR-CS, adrenal CS; ECT-CS, ectopic CS.

In the overall group, the median time from diagnosis to death was 9 (1-48) months. In the ECT-CS group that time was 2 (0.7-9) months, in PIT-CS it was 48 (30-102) months and in ADR-CS the time was known only in one patient who died (12 months) (**Figure 2**). Among ECT-CS patients the most prevalent cause of death was tumor progression without other identified risk factors (15 patients). Five patients died due to infections and tumor progression, two patients died during surgery, one patient died due to serious infection, one patient died due to PE, and in two cases the cause of death was not identified.

In the ADR-CS group, one patient died due to serious infection, one patient died during surgery, and in three cases the cause of death was not identified.

In the PIT-CS group, three patients died due to infection, one patient due to stroke, one patient due to pituitary tumor progression, and in two cases the cause of death was not identified.

The results are presented in **Table 5** and **Figure 2**.

**Table 5:** Mortality- in the overall population and each etiologic group in European Registry on Cushing’s syndrome (ERCUSYN) Krakow database.

Total	PIT-CS	ADR-CS	ECT-CS	Statistically significant differences
38/206 18%	7/113 6%	5/51 10%	26/42 62%	*** ECT-CS vs PIT CS ***ECT-CS vs ADR-CS

PIT-CS denotes pituitary CS; ADR-CS, adrenal CS; ECT-CS, ectopic CS. (***p<0.001).

## 4 Discussion

The study provides data on the mortality, baseline cardiometabolic comorbidities and venous thromboembolic events of a cohort of 214 patients with CS from a tertiary endocrinology center in Krakow, Poland, a part of the ERCUSYN database. Compared to the last official ERCUSYN analysis from 2022 (ERCUSYN 2022, entire database, N:1564), the proportion of ECT-CS was higher (22% vs 9%) in our centre (3). The explanation of this phenomenon could be associated with the center’s characteristic as a referral center receiving more complex and diagnostically challenging cases (26, 27). In our group the most common cause of ECT-CS was SCLC (20%), followed by lung carcinoid (16%), whereas in the overall database lung carcinoid (27%) was the most frequently reported tumor.

No inter-etiological difference in BMI, weight, or obesity prevalence was observed in the analyzed group, which was in line with the ERCUSYN 2022 results (3). However, anthropometric measures such as BMI have limited ability to reflect the metabolic phenotype of patients with Cushing’s syndrome. This limitation is driven by cortisol-related alterations in body composition, including enhanced cortisol action in visceral adipose tissue, increased 11β-HSD1–mediated cortisol regeneration, promotion of lipogenesis, insulin resistance, adipokine imbalance and sustained catabolic effects leading to progressive loss of skeletal muscle mass (3, 28, 29). These considerations are supported by findings from the population-based study by Sasson et al., which assessed patients with pituitary and adrenal Cushing’s syndrome (28). Our cohort additionally included patients with ectopic Cushing’s syndrome, who were not represented in the Sasson et al. analysis. ECT-CS typically presents with rapid onset, pronounced muscle wasting and significant fluid retention. As a result, BMI may appear artificially elevated due to water overload, whereas in other cases true underweight and severe wasting may be obscured by a seemingly normal BMI because of disproportionate loss of lean mass. Therefore, BMI is an unreliable indicator of nutritional status or metabolic burden in ectopic CS. Taken together, previously published data and our findings indicate that evaluation of metabolic burden in Cushing’s syndrome (particularly in ectopic cases), requires more refined assessment methods, including detailed body composition analysis or imaging-based evaluation of visceral adiposity, which better capture the severity of metabolic impairment than standard anthropometric indices. While not a perfect marker, waist circumference represents one of the simplest and most accessible clinical surrogates of visceral adiposity, potentially more informative than BMI. However, these data were not available in our cohort.

In our study, hypertension was the most common comorbidity. The prevalence is concordant with previous reports and is much higher than reported in the general non-CS population (30%) (3, 30). Patients with hypertension secondary to CS are estimated to be around 0-1% of cases overall and the prevalence increases up to 8% in patients with early-onset hypertension (31, 32). Cortisol overproduction causes hypertension via several mechanisms: mineralocorticoid agonist activity, increased vasoconstrictors production (angiotensinogen, endothelin I), greater vascular sensitivity to angiotensin II and catecholamines, decreased vasodilators production (prostacyclin, prostaglandins and kallikreins) and vascular remodeling promotion (31–33). Persistence of hypertension in 30% of cured patients is explained by irreversible vascular remodelling in cases of long lasting hypercortisolemia and hypertension (13, 31, 33). The impact of hypercortisolemia on the functional and structural changes in the vessels is also related with an increased risk of other cardiovascular complications (13). The data on the exact distribution of these comorbidities among CS patients is scarce. Based on one single-center study, the prevalence of myocardial infarct and stroke were respectively 4% and 5% (34), which in case of stroke was similar to our group. Based on literature, heart failure was suggested to be present in 1.7%-65.7% of CS patients, depending on the classification criteria (chronic/acute), while in our group the prevalence was 19% (5, 6, 35, 36). In the analyzed group, ischemic heart disease and heart failure were significantly more prevalent in ECT-CS as compared with PIT-CS, which may be associated with a more severe outcome of ECT-CS.

Glucose metabolism impairment in CS is a result of decreased insulin sensitivity and increased gluconeogenesis induced by cortisol excess (12). DM was present in 54%, IFG in 7%, and IGT in 6% of patients, which is comparable with previous studies. with higher DM prevalence than in the general population (10%) (3, 37–39). The prevalence of IGT and IFG are similar in the studied group compared to the general population (7%-IGT, 6%-IFG) (40, 41). Based on the ERCUSYN 2011 report, DM was significantly more prevalent in the ECT-CS group compared to two other groups, which is in line with our findings (24). These findings may be associated with a more severe course of hypercortisolemia in ECT-CS. In previous reports the prevalence of hypercortisolism in patients with diabetes was estimated to be around 6% overall, in patients with complications it was 10.5% and 2.0% in patients without complications (42). Recently published results of the CATALYST study, showed that the prevalence of hypercortisolism might be as high as 24% in patients with inadequately controlled DM (43, 44). However, these results should not be interpreted as reflecting the true prevalence of CS, given that the diagnostic approach relied mainly on the overnight 1-mg dexamethasone suppression test, which is prone to false-positive results in states of metabolic decompensation. Additional hormonal evaluations are required to distinguish CS from functional hypercortisolism. Nevertheless, CS prevalence might be encountered relatively frequent in patients with difficult to control DM, and a full diagnostic evaluation of hypercortisolemia should be considered in this population.

Although diabetes is not regarded as a risk factor for VTE, diabetes and CS share underlying contributors to the prothrombotic state, such as enhanced thrombin generation, a proinflammatory state, and increased release of neutrophil extracellular traps (45, 46). Moreover, patients with CS have an increased concentration of prothrombotic factors such as von Willebrand factor, fibrinogen and factor-VIII, resulting in a markedly higher risk of VTE events (47). According to the ERCUSYN 2024 report on VTE events, the prevalence was 4.4%, the cases were predominated by PIT-CS patients and male gender was a risk factor for VTE events (19). Based on other studies, the prevalence was 2.7%-9.6% (48, 49). A previous report from our center performed on a smaller CS group, showed a prevalence of 7.8% (50). The present study estimated a high prevalence of VTE events, occurring prior to endocrine evaluation (N:12; 6%). In three of these patients, VTE served as the primary indication for referral to an endocrinologist (51, 52). Based on other studies, the pre-diagnostic VTE risk in CS patients was 0.9-4% (49, 50, 53).

Hypercortisolemia has a detrimental impact on health overall. Due to many comorbidities secondary to hypercortisolemia, patients with CS have an increased mortality rate not only at the time of active CS, but also after the cure (54–59). According to recent data analyzing long-term mortality of CS and the ERCUSYN 2019 report which analyzed short term mortality of CS, the most prevalent causes of death were infectious diseases (28.6%-31%) (21, 59). Previous studies indicated that cardiovascular diseases contributed mostly to the CS mortality (6). In our analysis, the ECT-CS group showed a significantly higher mortality rate than other groups, which is in line with the ERCUSYN 2019 report (21). The most common cause of death was tumor progression and infectious disease, which was in line with other reports (21, 59). Mortality rate was significantly higher among men than women, which was coherent with recent data (59). That can be explained by the greater proportion of ECT-CS among males than females.

The main limitations of this study is retrospective and monocentric design. Moreover, the data on the etiology distribution in the study might be influenced by the tertiary character of our center, where more complicated and challenging cases are referred. Thus, the percentage of diagnosed and reported ectopic CS is higher in our study compared to other reports. The lack of follow-up data on comorbidities and the absence of additional analyses evaluating factors influencing mortality represent another limitation of this study. The strength of the study is the quality of analyzed data and the complexity of the analysis which involved baseline patients’ characteristics as well as detailed data on patients’ comorbidities. Moreover, the care of patients from the moment of diagnosis through the follow up process, was performed by the same pituitary center, specialized in the diagnosis and management of CS patients.

## 5 Conclusions

The prevalence of ECT-CS in our study was higher than in other reports, which is related to the specificity of our tertiary center. CS patients from our study presented a high number of comorbidities and high mortality. The ECT-CS group showed the highest prevalence of DM, heart failure, ischemic heart disease, and higher mortality rate, which may correspond with a more severe course of hypercortisolemia in this group. The majority of the results are convergent with the entire ERCUSYN database reports and data from the literature.

## Data Availability

The raw data supporting the conclusions of this article will be made available by the authors, without undue reservation.
